# Tau positron emission tomography harmonization using the CenTauR approach: General conversion equations, relevant cut‐points, and external validation

**DOI:** 10.1002/alz.71886

**Published:** 2026-10-30

**Authors:** Alexis Moscoso Rial, Antoine Leuzy, Lars Lau Raket, Victor L. Villemagne, Gregory Klein, Matteo Tonietto, Emily Olafson, Ruben Smith, Christopher G. Schwarz, Suzanne Baker, Ziad S. Saad, Santiago Bullich, Brian Lopresti, Sandra Sanabria Bohorquez, Guilherme Domingues Kolinger, Olivia Lutz, Mercè Boada, Tobey J. Betthauser, Arnaud Charil, Emily C. Collins, Jessica A. Collins, Roger N. Gunn, Makoto Higuchi, Eric Hostetler, R. Matthew Hutchison, Leonardo Iaccarino, Philip S. Insel, Michael C. Irizarry, Clifford R. Jack, William J. Jagust, Keith A. Johnson, Sterling C. Johnson, Cécile Tissot, David Vállez García, Gemma Salvadó, Diane Stephenson, Marta Marquié, Sulantha Mathotaarachchi, Mark A. Mintun, Rik Ossenkoppele, Qi Huang, Xiaoxie Mao, Johannes Gnörich, Ioannis Pappas, Ronald C. Petersen, Konstantinos Chiotis, Gil D. Rabinovici, Pedro Rosa‐Neto, Andrew W. Stephens, Alex Whittington, Maria C. Carrillo, Michael J. Pontecorvo, Samantha Budd Haeberlein, Billy Dunn, Hartmuth C. Kolb, Nadine Tatton, Yashmin Karten, Matthias Brendel, Sid O'Bryant, Fang Xie, Christopher C. Rowe, Oskar Hansson, Vincent Doré, for the Alzheimer's Disease Neuroimaging Initiative (ADNI), for the Health and Aging Brain Study (HABS‐HD) Study Team

**Affiliations:** ^1^ ZRO Imaging Santiago de Compostela Spain; ^2^ Nuclear Medicine Department and Molecular Imaging Group Instituto de Investigación Sanitaria de Santiago de Compostela Santiago de Compostela Spain; ^3^ Eli Lilly and Company Indianapolis Indiana USA; ^4^ Department of Psychiatry University of Pittsburgh School of Medicine Pittsburgh Pennsylvania USA; ^5^ Department of Molecular Imaging & Therapy Austin Health, Melbourne Victoria Australia; ^6^ F. Hoffmann‐La Roche Ltd Basel Switzerland; ^7^ Clinical Imaging Group Genentech, Inc. South San Francisco California USA; ^8^ Clinical Memory Research Unit, Department of Clinical Sciences Lund University Lund Sweden; ^9^ Memory Clinic Skåne University Hospital Malmö Sweden; ^10^ Department of Radiology Mayo Clinic Rochester Minnesota USA; ^11^ Lawrence Berkeley National Laboratory University of California Berkeley Berkeley California USA; ^12^ Johnson & Johnson La Jolla California USA; ^13^ Life Molecular Imaging GmbH Berlin Germany; ^14^ Department of Radiology University of Pittsburgh Pittsburgh Pennsylvania USA; ^15^ Lantheus Switzerland GmbH Basel Switzerland; ^16^ Ace Alzheimer Center Barcelona Barcelona Spain; ^17^ Networking Research Center on Neurodegenerative Diseases (CIBERNED) Madrid Spain; ^18^ Wisconsin Alzheimer's Disease Research Center University of Wisconsin– Madison School of Medicine and Public Health Madison Wisconsin USA; ^19^ Department of Medicine Division of Geriatrics University of Wisconsin–Madison School of Medicine and Public Health Madison Wisconsin USA; ^20^ Department of Medical Physics University of Wisconsin–Madison Madison Wisconsin USA; ^21^ Eisai, Inc. Nutley New Jersey USA; ^22^ Biogen, Inc. Cambridge Massachusetts USA; ^23^ Xing Imaging – AMitro Company London UK; ^24^ Advanced Neuroimaging Center, Institute for Quantum Medical Science National Institutes for Quantum Science and Technology Chiba Japan; ^25^ Merck & Co. West Point Pennsylvania USA; ^26^ Department of Psychiatry and Behavioral Sciences University of California San Francisco California USA; ^27^ Harvard Medical School Department of Radiology Boston Massachusetts USA; ^28^ Gordon Center for Medical Imaging Massachusetts General Hospital Boston Massachusetts USA; ^29^ Barcelonaβeta Brain Research Center Barcelona Spain; ^30^ Centro de Investigación Biomédica en Red de Bioingeniería Biomateriales y Nanomedicina (CIBER‐BBN), Instituto de Salud Carlos III Madrid Spain; ^31^ Critical Path for Alzheimer's Disease (CPAD) Consortium Critical Path Institute Tucson Arizona USA; ^32^ Enigma Biomedical Group Knoxville Tennessee USA; ^33^ Neuroscience Campus Amsterdam, Alzheimer center Amsterdam University Medical Center Amsterdam The Netherlands; ^34^ Department of Nuclear Medicine & PET Center Huashan University Hospital Shanghai China; ^35^ Department of Nuclear Medicine, LMU Hospital Ludwig Maximilian University of Munich Munich Germany; ^36^ German Center for Neurodegenerative Diseases (DZNE) Munich Munich Germany; ^37^ Laboratory of Neuro Imaging, USC Stevens Neuroimaging and Informatics Institute, Keck School of Medicine of USC University of Southern California Los Angeles California USA; ^38^ Department of Neurology Mayo Clinic Rochester Minnesota USA; ^39^ Center of Alzheimer Research, Division of Clinical Geriatrics, Department of Neurobiology Care Sciences and Society, Karolinska Institutet Stockholm Sweden; ^40^ Department of Neurology, Edward and Pearl Fein Memory and Aging Center, Weill Institute for Neurosciences University of California San Francisco California USA; ^41^ Department of Radiology & Biomedical Imaging University of California, San Francisco San Francisco California USA; ^42^ Translational Neuroimaging Laboratory, Department of Neurology and Neurosurgery The McGill University Research Center for Studies in Aging, Montreal Neurological Institute, McGill University Montreal Canada; ^43^ Peter O'Donnell Jr. Brain Institute (OBI) University of Texas Southwestern Medical Centre Dallas Texas USA; ^44^ Perceptive Discovery London UK; ^45^ Alzheimer's Association Chicago Illinois USA; ^46^ Senior advisor to CPAD Consortium Critical Path Institute Tucson Arizona USA; ^47^ Munich Cluster for Systems Neurology (SyNergy) Munich Germany; ^48^ Institute for Translational Research University of North Texas Health Science Center Fort Worth Texas USA; ^49^ Florey Department of Neuroscience University of Melbourne Melbourne Victoria Australia; ^50^ The Australian Dementia Network (ADNeT) The University of Melbourne Melbourne Victoria Australia; ^51^ Health and Biosecurity Flagship The Australian eHealth Research Centre, CSIRO Melbourne Victoria Australia

**Keywords:** Alzheimer's disease, biomarkers, CenTauR, harmonization, Joint Propagation Model, positron emission tomography imaging, radiotracer standardization, standardized uptake value ratio, tau positron emission tomography

## Abstract

**INTRODUCTION:**

Tau positron emission tomography (PET) tracers differ in kinetics, binding properties, and quantification pipelines, complicating cross‐study comparisons. The CenTauR approach provides a unified framework for harmonizing tau PET quantification across tracers.

**METHODS:**

We defined a tracer‐agnostic “Standard Method” using Statistical Parametric Mapping 12, standardized regions of interest, and harmonized acquisition parameters. Using data from five tau tracers—[^18^F]flortaucipir, [^18^F]MK‐6240, [^18^F]PI‐2620, [^18^F]RO948, and [^18^F]GTP1—we applied the Joint Propagation Model to derive linear equations converting regional standardized uptake value ratios into the tracer‐independent CenTauR scale. Validation included independent cross‐sectional, longitudinal, and test–retest datasets across multiple cohorts.

**RESULTS:**

The CenTauR scale yielded highly consistent regional values across tracers, with strong cross‐tracer agreement and robust performance in external validation cohorts. We also expressed clinically relevant cut‐points and staging boundaries on the CenTauR scale.

**DISCUSSION:**

The CenTauR approach enables harmonized, tracer‐independent quantification of tau PET, supporting reproducibility across studies and improving interpretation in clinical research and multicenter trials.

## Highlights

- CenTauR enables harmonized tau positron emission tomography quantification across tracers.
- The Joint Propagation Model derives tracer‐independent conversion to a unified CenTauR scale.
- The standard method defines Statistical Parametric Mapping 12–based standardized uptake value ratio for harmonization.
- CenTauR cut‐points enable consistent staging and positivity definition
- External validation shows consistent results across matched cohorts.

## 1 BACKGROUND

Tau positron emission tomography (PET) has rapidly evolved over the past decade, enabling accurate in vivo visualization of tau neurofibrillary tangle pathology in Alzheimer's disease (AD).^1^, ^2^, ^3^ The first‐generation tau PET tracer [^18^F]flortaucipir ([^18^F]AV‐1451, [^18^F]T‐807), now approved for clinical use by both the US Food and Drug Administration (FDA), the European Medicines Agency, and the Medicines and Healthcare products Regulatory Agency (MHRA) in the UK, among others, has demonstrated high accuracy for detecting and monitoring tau deposition.^4^, ^5^, ^6^, ^7^, ^8^, ^9^, ^10^, ^11^, ^12^, ^13^, ^14^, ^15^ In addition, several novel tau PET radiotracers, including [^18^F]florquinitau (also known as [^18^F]MK‐6240), [^18^F]PI‐2620, [^18^F]RO948, [^18^F]GTP‐1, and [^18^F]florzolotau ([^18^F]APN‐1607) have been developed, offering different dynamic ranges and off‐target binding profiles.^16^, ^17^, ^18^, ^19^, ^20^, ^21^ The approval and growing availability of tau PET radiotracers has expanded tau research and facilitated the use of tau PET in clinical practice and trials. However, differences in tracer binding properties and analytical methodologies pose challenges for standardizing tau PET quantification and ensuring comparability across different clinical studies and trials, and precludes the use of different tau PET tracers within a single trial.

Building on the same principles that enabled the successful harmonization of amyloid PET tracers and the widespread adoption of the Centiloid scale,^22^ the Critical Path for Alzheimer's Disease Consortium (CPAD) at the Critical Path Institute (C‐Path) convened a working group of experts from industry and academia to address the tau PET harmonization challenge. The working group has previously proposed the use of standardized regions of interest (ROIs) for tau PET quantification, termed the “Standard CenTauR” ROIs.^23^ These ROIs, together with a recently released tau PET quantification pipeline (https://www.gaain.org/centaur‐project), were developed to provide a unified framework for tau PET analysis, improving comparability and reproducibility across studies. In parallel, the working group also developed the Joint Propagation Model (JPM), a statistical approach that harmonizes standardized uptake value ratios (SUVRs) from different tracers into a single, tracer‐independent metric—the CenTauR scale.^24^ Unlike traditional methods relying on a single reference tracer, the JPM simultaneously models the relationships between multiple tracers, anchor points, and test–retest tau PET data, reducing conversion error and bias compared to linear regression methods based on a reference tracer. Together, these advances demonstrate the feasibility of a CenTauR‐based strategy for reliable tau PET harmonization.

Here, we hypothesized that tau PET measurements obtained using different radiotracers and quantification pipelines could be transformed onto a common, biologically anchored scale. To test this hypothesis, we built on previous efforts of the CPAD consortium and described the CenTauR harmonization approach, comprising: (1) a “Standard Method” for tau PET quantification that establishes a common reference SUVR scale; (2) the JPM, which provides linear equations for converting “standard” SUVRs into CenTauR units; and (3) a calibration procedure for tau PET metrics obtained using non‐standard methods. We also provide clinically relevant tau PET cut‐points on the CenTauR scale for visual‐read positivity, biological staging, and tau‐load stratification. Finally, we evaluated the external validity of the approach by comparing CenTauR‐defined tau PET positivity frequencies across clinically and demographically matched cohorts scanned with different radiotracers and by benchmarking its performance against the *z* score–based CenTauR_z_ method.^23^ Overall, this article is intended to serve as a practical guide for implementing the CenTauR framework, while providing relevant cut‐points and an initial validation of its performance.

**RESEARCH IN CONTEXT**

1. **Systematic review**: We reviewed prior work on tau positron emission tomography (PET) harmonization, including CenTauR regions of interest, the Joint Propagation Model (JPM), and related tracer comparison studies using PubMed and conference proceedings. Existing studies highlight variability across tracers and pipelines, but lack a unified, validated framework for cross‐tracer standardization.
2. **Interpretation**: We present the CenTauR approach as a complete harmonization framework, integrating a standardized quantification method with JPM‐based conversion equations. Our findings demonstrate that tau PET measures can be expressed on a common, tracer‐independent scale with consistent cut‐points and improved comparability across cohorts and tracers.
3. **Future directions**: Future work should validate CenTauR in additional tracers and longitudinal datasets, assess sensitivity to acquisition and reconstruction differences, and refine biologically grounded cut‐points through PET‐to‐autopsy studies. Further evaluation in clinical trials is needed to establish its utility for treatment monitoring and patient stratification.

## 2 THE CenTauR APPROACH

Building on the success of the Centiloid scale^22^ in harmonizing amyloid beta (Aβ) PET quantitative measures across different tracers and methodologies, we designed the CenTauR approach using similar fundamental principles. Importantly, given the absence of a clear gold‐standard tau PET tracer, we moved beyond the Centiloid framework's reliance on a single reference tracer by using the JPM for between‐tracer harmonization. In the following sections, we describe the building blocks of the CenTauR harmonization approach, namely the “Standard Method” for obtaining Standard SUVR data from tau PET images, and the JPM‐based equations for conversion of Standard SUVR data into the CenTauR scale (Figure 1).

**FIGURE 1:**
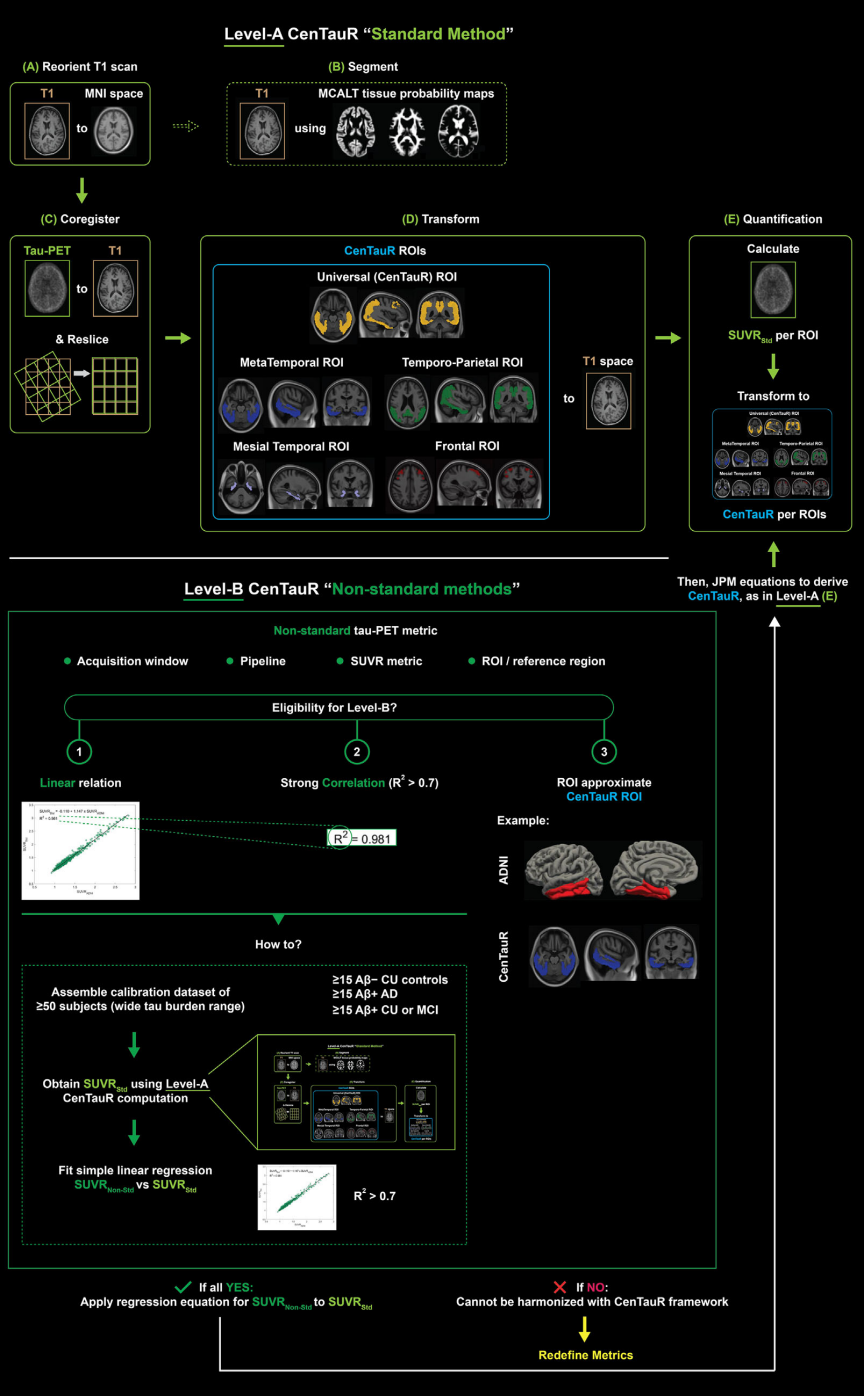
Schematic representation of the CenTauR harmonization approach. Top panel (Level A), Standardized pipeline for direct CenTauR computation. A, T1‐weighted MRI is reoriented and normalized to MNI space. B, Tissue segmentation is performed in SPM12 using MCALT tissue probability maps. C, Tau PET images are rigidly coregistered to the subject's T1 MRI and resliced. D, Standard CenTauR ROIs—including Universal, Meta Temporal, Mesial Temporal, Temporoparietal, and Frontal—are transformed into native T1 space. E, SUVR_Std_ values are computed within each ROI using an inferior cerebellar gray matter reference region and converted to CenTauR units using tracer‐specific JPM equations. Bottom panel (Level B), Calibration framework for non‐standard tau PET metrics. Non‐standard measures (e.g., alternative acquisition windows, processing pipelines, SUVR definitions, or ROIs) are evaluated for eligibility based on (1) approximate linearity, (2) strong correlation with SUVR_Std_ (*R* ^2^ > 0.7), and (3) anatomical correspondence with CenTauR ROIs. If criteria are met, a linear regression is derived using a calibration dataset spanning a wide range of tau burden, enabling transformation of non‐standard metrics to SUVR_Std_ and subsequently to CenTauR units. If criteria are not satisfied, the metric cannot be harmonized within the CenTauR framework. Aβ, amyloid beta; ADNI, Alzheimer's Disease Neuroimaging Initiative; CU, cognitively unimpaired; JPM, Joint Propagation Model; MCALT, Mayo Clinic Adult Lifespan Template; MCI, mild cognitive impairment; MNI, Montreal Neurological Institute; MRI, magnetic resonance imaging; PET, positron emission tomography; ROI, region of interest; SPM, Statistical Parametric Mapping; SUVR, standardized uptake value ratio.

### 2.1 The Standard Method

The numerical value of tau PET quantification results can be highly influenced by various technical and methodological factors beyond the radiotracer, especially the quantification pipeline used.^25^, ^26^ To remove pipeline‐driven systematic differences and enable robust between‐tracer harmonization, we first define a “Standard Method”—the reference method for tau PET image acquisition and quantification—whose SUVR units serve as the common scale for subsequent between‐tracer harmonization. Below, we outline the specific steps for implementing the Standard Method in the CenTauR framework, including precise definitions of Standard scanning time windows, the tau PET quantification pipeline, and the brain ROIs where tau signal is measured.

#### 2.1.1 Scanning time window

Given that tau PET quantification is sensitive to the acquisition time window,^18^, ^19^, ^27^, ^28^, ^29^ we define Standard scanning time windows for each tau PET radiotracer based on either its FDA‐approved label ([^18^F]flortaucipir^30^) or the most commonly used time window in previous studies^31^, ^32^, ^33^ (Table S1 in supporting information). We note that tau PET scans acquired using non‐Standard scanning windows, that is, scanning windows different from the Standard ones defined in Table S1, cannot be used to directly measure Standard SUVR within the framework of the Standard Method but can still be harmonized using the CenTauR approach (see Section 3.2).

#### 2.1.2 Quantification pipeline

We adopted a Statistical Parametric Mapping (SPM)‐based quantification pipeline given its proven success and widespread adoption as the standard quantification pipeline for the Centiloid scale. However, rather than using the original Centiloid pipeline implemented in SPM8,^22^ we updated it to a more recent SPM12‐based version, which provides an improved balance of accuracy, reliability, and usability. SPM12's tissue segmentation and spatial normalization are now widely adopted for PET quantification in AD research, facilitating seamless integration of the CenTauR Standard Method into existing workflows. The CenTauR SPM12‐based pipeline computes SUVR directly in magnetic resonance imaging (MRI) space using tissue‐class masking and the population‐matched Mayo Clinic Adult Lifespan Template (MCALT) priors (https://www.nitrc.org/projects/mcalt/). Specifically, the pipeline rigidly coregisters and resamples the static (frame‐averaged or purely static acquisition) tau PET to the subject's T1 MRI, segments the MRI with SPM12's Unified Segmentation algorithm using MCALT priors, and resamples the Standard CenTauR ROIs (see next section) in MRI space. The CenTauR framework assumes a single‐frame PET image as input; the choice between static or frame‐averaged data, as well as the realignment approach, is left to the user and is not expected to have a major impact on harmonization. SUVRs are then calculated by measuring signal within the intersection of each CenTauR ROI and brain parenchyma (tissue mask c1 + c2 ≥ 0.5, i.e., gray matter [GM] + white matter [WM]) and dividing by the signal in an inferior cerebellar GM reference ROI.^23^

We note that the purpose of this pipeline is to establish a common reference SUVR scale for subsequent between‐tracer harmonization, rather than to replace existing pipelines that may be better suited to specific research or clinical applications. Importantly, CenTauR harmonization is intended to preserve the properties of these alternative pipelines by linearly rescaling their metrics onto a common unit, as described in Section 3.2. Accordingly, pipeline‐specific variability is not expected to disappear after calibration, while enabling these measures to be interpreted consistently across tracers and quantification pipelines.

#### 2.1.3 Brain ROIs

A predefined set of Standard CenTauR ROIs^23^ (https://gaain.org/centaur‐project)—including an inferior cerebellum GM reference region common to all tau PET tracers—is used for SUVR quantification in the Standard Method. The inferior cerebellar GM reference region is derived from the Centiloid cerebellar cortex mask, with the superior portion excluded to minimize off‐target binding and the inferior portion excluded to avoid quantification problems.^23^ The five target ROIs include the Universal ROI, as well as its four component subregions: Meta Temporal, Mesial Temporal, Temporoparietal, and Frontal. This panel of target ROIs provides users with different options for assessing tau burden, either through measures that are more descriptive of tau PET positivity status or global tau load (Meta Temporal and Universal ROIs) or through topography‐based tau staging systems (Mesial Temporal, Temporoparietal, and Frontal ROIs). The Universal ROI was designed to capture relevant overlapping signal across radiotracers while maximizing discrimination between Aβ‐positive AD dementia and Aβ‐negative controls and minimizing tracer‐specific off‐target signal,^23^ thus maximizing suitability for harmonization. Additional details of the Standard CenTauR ROIs are provided in Villemagne et al.^23^

Hereafter, SUVR values measured using the Standard Method outlined above are referred to as Standard SUVR (SUVR_Std_), and their computation is termed Level‐A CenTauR computation (see Section 3.1).

### 2.2 The JPM

After standardizing tau PET quantification using the Standard Method, a between‐tracer harmonization strategy can be applied to enable quantitative comparisons across different radiotracers. Within the CenTauR approach, this between‐tracer harmonization is achieved using the JPM.^24^ The JPM integrates all the available head‐to‐head (tau PET scans acquired with different tracers in the same individuals), anchor point (acquired in predefined groups that anchor the scale at 0 and 100; see below), and test–retest (repeated scans with the same tracer in the same subjects) tau PET SUVR_Std_ data from multiple tracers within a unified statistical framework, eliminating the need for a single reference tracer. Specifically, for tracer t and subject i, the specific JPM model used in the CenTauR framework can be written as

$$\;\mathrm{SUV}R_{it}=a_{t}\;\left[1_{H2H\left(i\right)}c_{i}+1_{\mathrm{TRT}\left(i\right)}c_{i}+1_{\mathrm{CTR}-0\left(i\right)}c_{i}^{0}+1_{\mathrm{CTR}-100\left(i\right)}c_{i}^{100}\right]+b_{t}+\varepsilon_{it},$$

where $a_{t}$ and $b_{t}$ are tracer‐specific slope and intercept parameters, respectively; $c_{i}$ are modeled as fixed effects identical across tracers, while $c_{i}^{0}∼N\left(0,\sigma_{0}^{2}\right)$ and $c_{i}^{100}∼N\left(100,\sigma_{0}^{2}\right)$ are Gaussian‐distributed random effects centered at 0 and 100, respectively, with variance $\sigma_{0}^{2}$; $1_{H2H(i)}$, $1_{\mathrm{TRT}(i)},\;1_{\mathrm{CTR}-0(i)}$, and $1_{\mathrm{CTR}-100(i)}$ are indicator functions that are 1 if subject i belongs to the head‐to‐head, test–retest, CTR‐0, or CTR‐100 datasets, respectively, and 0 otherwise; and $\varepsilon_{it}$ is a Gaussian‐distributed heteroscedastic error term that assumes different variance across tracers in the CenTauR scale, that is,

$$\varepsilon_{it}∼N\left(0,a_{t}^{2}\sigma {\left(c_{i}\right)}^{2}\right),$$

where

$$\sigma \;\left(c_{i}\right)=\sigma_{1}+\sigma_{2}{\left|c_{i}\right|}^{p}$$

In the JPM, tracer‐to‐tracer relationships are modeled assuming linear relationships. To assess whether a more complex functional form was necessary, we performed a sensitivity analysis comparing the linear model with a smooth‐transition piecewise linear regression using a hyperbolic tangent function. Model fit was compared using the Akaike information criterion (AIC). Across tracer–region combinations, the piecewise model did not show a consistent improvement over the linear model and, in some cases, showed evidence of overfitting at the upper end of the SUVR range (Figure S1 in supporting information). Based on these findings, a functional linear form was retained for modeling tracer relationships within the JPM in the CenTauR framework. The JPM output is a set of linear equations for converting SUVR_Std_ values to CenTauR units (CTR), which is anchored at CTR = 0 (CTR‐0)—corresponding to the average value of a group of Aβ PET–negative (visual read and Centiloid < 10), tau PET–negative (visual read^24^), cognitively unimpaired (CU) controls—and CTR = 100 (CTR‐100)—corresponding to the average value of Aβ PET–positive (visual read and Centiloid > 40, except for participants from the second Biomarkers For Identifying Neurodegenerative Disorders Early and Reliably (BioFINDER‐2) study, in whom Aβ positivity was established using cerebrospinal fluid Aβ_42_/Aβ_40_), tau PET–positive (visual read) AD dementia patients < 75 years of age who have a Mini‐Mental State Examination (MMSE) score ≥ 20. A description of the anchor point datasets used to fit the JPM is provided below. While the JPM results in the same type of simple linear conversion equations as standard linear regression methods, this method has demonstrated superior accuracy in harmonizing tau PET data.^24^

To derive general conversion equations for the CenTauR scale, we applied the Standard Method to process images from 116 participants scanned head‐to‐head with two different tau PET tracers and 367 anchor‐point participants. The head‐to‐head dataset included the following cohorts: [^18^F]flortaucipir versus [^18^F]MK‐6240 (University of Pittsburgh, *n *= 15),^34^ [^18^F]flortaucipir versus [^18^F]RO948 (BioFINDER‐2, *n* = 34),^35^ [^18^F]MK‐6240 versus [^18^F]GTP1 (Roche/Invicro, *n* = 22),^36^ [^18^F]PI‐2620 versus [^18^F]GTP1 (Roche/Invicro, *n* = 28),^36^ and [^18^F]PI‐2620 versus [^18^F]RO948 (Fundació ACE Healthy Brain Initiative [FACEHBI] study, *n* = 17).^37^ The anchor point dataset included subjects scanned with [^18^F]flortaucipir (*n* = 59 CTR‐0, *n* = 31 CTR‐100 [Avid A05]), [^18^F]MK‐6240 (*n* = 94 CTR‐0, *n* = 63 CTR‐100 [Australian Imaging, Biomarker and Lifestyle study]), [^18^F]PI‐2620 (*n* = 10 CTR‐0, *n* = 7 CTR‐100 [Life Molecular Imaging, Alzheimer's Disease Neuroimaging Initiative (ADNI)], [^18^F]RO948 (*n* = 34 CTR‐0, *n* = 29 CTR‐100 [BioFINDER‐2]), [^18^F]GTP1 (*n* = 15 CTR‐0, *n* = 25 CTR‐100 [Roche/Invicro]; see Table S2 in supporting information for dataset‐specific details). Additional demographic and biomarker information of these cohorts can be found in Tables S3 and S4 in supporting information. As required by the Standard Method, all tau PET scans were acquired using a standard scanning time window (Table S1), except for the [^18^F]MK‐6240 scans from the University of Pittsburgh, that were acquired using a non‐standard window of 70 to 90 minutes (Standard window for [^18^F]MK‐6240 is 90–110 minutes). To make this [^18^F]MK‐6240 SUVR data compatible with the Standard Method, we used a subset of 76 participants from the National Alzheimer's Coordinating Center (NACC) dataset who had [^18^F]MK‐6240 SUVR measured with both 70 to 90 minutes and 90 to 110 minute scanning windows to derive a linear conversion equation transforming the non‐standard (70–90 minutes) SUVR into SUVR_Std_ (90–110 minutes; Figure S2 in supporting information; *R* ^2^ > 0.99 for the correlations between non‐standard SUVR and SUVR_Std_). These converted SUVR_Std_ values were then integrated with those from the remaining standardly acquired tau PET scans. Finally, a total of 77 subjects with test–retest tau PET scans ([^18^F]flortaucipir, *n *= 21 [time between scans: between 48 hours and 4 weeks]; [^18^F]MK‐6240, *n *= 15 [between 72 hours and 3 weeks]; [^18^F]PI‐2620, *n *= 12 [within 3 weeks]; [^18^F]RO948, *n* = 9 [between 14 to 38 days]; [^18^F]GTP‐1, *n* = 20 [between 3 and 25 days]; see Table S2 for dataset‐specific details; see Figure S3 and Table S5 in supporting information for additional details) was included in the model similarly to head‐to‐head data, for which each pair of observations was treated as separate observations performed with the same tracer within a subject (i.e., same true CenTauR value). Inclusion of test–retest data allowed for the estimation of tracer‐specific differences in variance parameters.^24^ The JPM was fitted on the combined dataset, yielding the general equations for conversion of SUVR_Std_ into the CenTauR scale (Table 1). The resulting CenTauR values for the head‐to‐head datasets (Figure 2) demonstrated high harmonization accuracy, with mean absolute differences (defined as $\frac{1}{n}\sum_{i}\left|CTR_{a}-CTR_{b}\right|$, where $\mathrm{CT}R_{a}$ and $\mathrm{CT}R_{b}$ are the estimated CenTauR values for tracers *a* and *b*, respectively, and the sum extends over all subjects) of 13.3 CenTauRs for the Meta‐Temporal region, 13.0 for Universal, 15.6 for Mesial Temporal, 12.7 for Temporoparietal, and 15.9 for Frontal regions. Similarly, CTR‐0 and CTR‐100 anchor‐point cohorts showed CenTauR distributions appropriately centered at 0 and 100 CTR, respectively (Figure 3).

**TABLE 1:** General equations for conversion of Standard SUVR (SUVR_Std_) into the CenTauR scale, for each Standard CenTauR region of interest.

Radiotracer	Meta Temporal	Universal	Mesial Temporal	Temporoparietal	Frontal
[^18^F]Flortaucipir	101.61 × SUVR_Std_−122.2	103.91 × SUVR_Std_−123.54	118.26 × SUVR_Std_−134.57	99.84 × SUVR_Std_−119.27	128.97 × SUVR_Std_−146.64
[^18^F]MK‐6240	42.47 × SUVR_Std_−43.07	44.39 × SUVR_Std_−43.13	47.31 × SUVR_Std_−40.64	42.11 × SUVR_Std_−42.43	56.46×SUVR_Std_−48.88
[^18^F]PI‐2620	97.25 × SUVR_Std_−108.68	98.9 × SUVR_Std_−105.7	115.87 × SUVR_Std_−121.61	94.09 × SUVR_Std_−100.56	101.44×SUVR_Std_−99.73
[^18^F]RO948	77.38 × SUVR_Std_−91.19	78.86 × SUVR_Std_−92.49	86.23 × SUVR_Std_−91.88	76.26 × SUVR_Std_−90.38	107.87×SUVR_Std_−119.12
[^18^F]GTP‐1	125.09 × SUVR_Std_−147.33	132.08 × SUVR_Std_−149.97	145.54 × SUVR_Std_−165.87	125.49 × SUVR_Std_−144.13	150.99 × SUVR_Std_−159.01

Abbreviations: PET, positron emission tomography; SUVR, standardized uptake value ratio.

**FIGURE 2:**
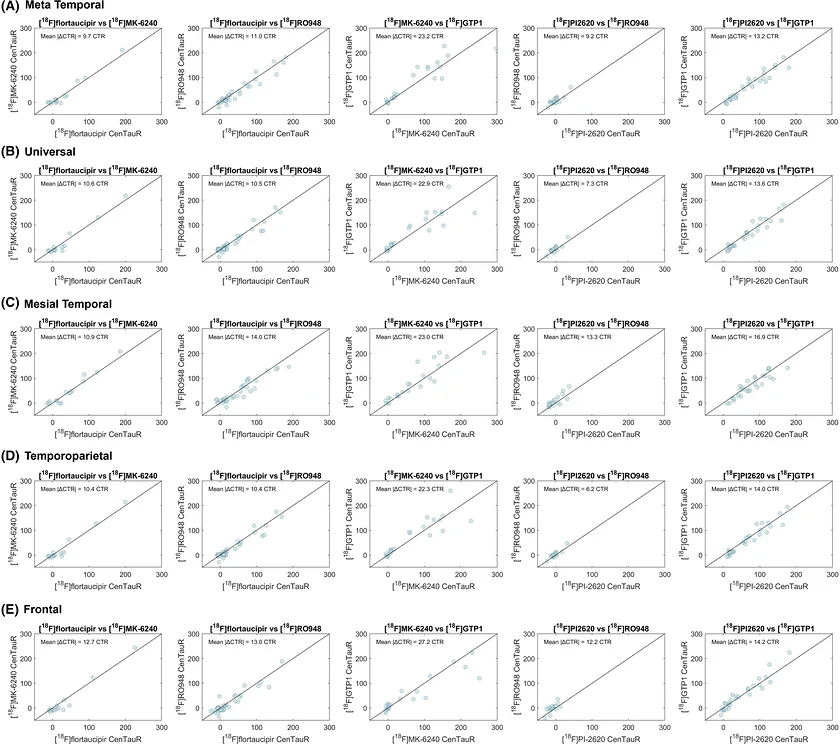
Relationship between JPM‐derived CenTauR values across five datasets comprising participants scanned head‐to‐head with two different tau PET tracers, shown for the various Standard CenTauR regions of interest. Δ|CTR| denotes the mean absolute difference (defined as $\frac{1}{n}\sum_{i}\left|CTR_{a}-CTR_{b}\right|$, where $\mathrm{CT}R_{a}$ and $\mathrm{CT}R_{b}$ are the estimated CenTauR values for tracers *a* and *b*, respectively) between each pair of corresponding CenTauR values. Red dashed lines indicate the linear regression fits, and the black line represents the identity line. JPM, Joint Propagation Model; PET, positron emission tomography.

**FIGURE 3:**
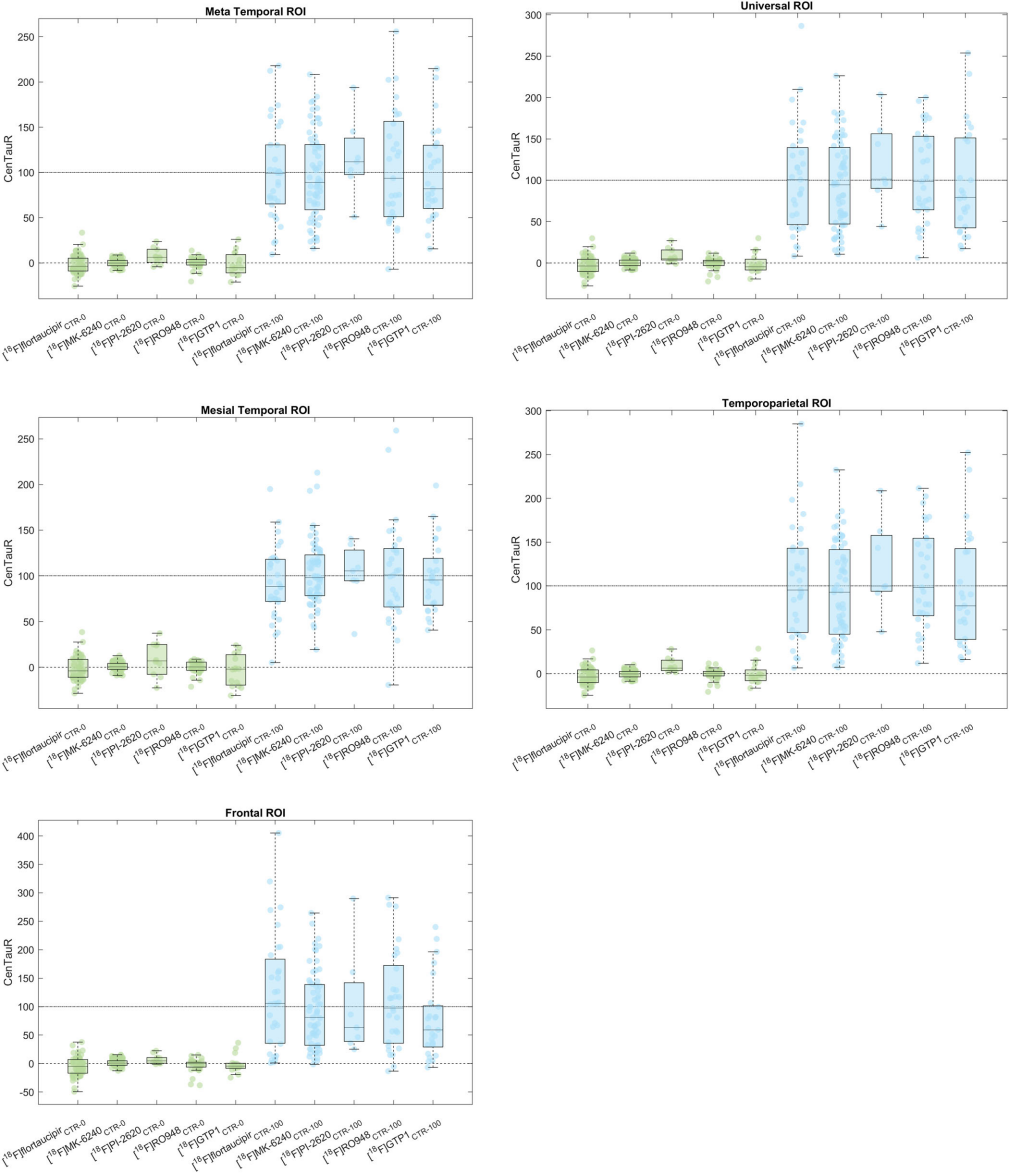
CenTauR values across the CenTauR regions of interest in the anchor point datasets. Green boxes represent subjects anchored to a CenTauR value of 0, while blue boxes represent subjects anchored to a CenTauR value of 100. ROI, region of interest.

## 3 COMPUTING CenTauRs

In this section, we describe the steps for computing CenTauR values based on either the Standard Method or based on tau PET SUVR data measured using non‐standard methods. These approaches correspond to Level 3 and Level 2 processes, respectively, in the Centiloid framework,^22^ which we redefine here as Level A (i.e., Standard SUVR) and Level B (i.e., non‐standard SUVR) CenTauR computation to avoid confusion between the CenTauR and Centiloid scales.

### 3.1 Level A CenTauR computation

The Level A CenTauR computation, analogous to the Level 3 process for the Centiloid scale,^22^ involves an exact replication of the steps detailed in Section 2—namely, SUVR quantification using the Standard Method and application of the JPM conversion equations from Table 1. To ensure seamless and reproducible Level A CenTauR computation, we provide publicly available code on the Global Alzheimer's Association Interactive Network (GAAIN) website that implements both the Standard Method and the JPM conversion equations (https://www.gaain.org/centaur‐project). This pipeline takes an individual's tau PET scan and corresponding T1 MRI as inputs and provides SUVR_Std_ and CenTauR values across the five Standard CenTauR ROIs. The code is available both as a MATLAB script as well as a standalone executable. As with any neuroimaging processing pipeline, visual inspection ensuring correct coregistration between the tau PET and T1‐weighted MRI scan, as well as appropriate spatial normalization, is required.

While we recommend using the provided GAAIN code for Level A CenTauR computation, individual sites may choose to code their own pipelines replicating the Standard Method's SPM12‐based pipeline and apply conversion equations from Table 1. In such cases, sites must validate their in‐house developed Standard pipeline by demonstrating a strong identity linear correlation (in CenTauR units, acceptable agreement is defined by a slope between 0.98 and 1.02, an intercept between −2 and 2 CTR, and an *R* ^2^ > 0.98) between their measured SUVR_Std_ results and those generated with the pipeline provided on the GAAIN website. These thresholds for validity are identical to those established for the Centiloid scale, which allow for the small degree of unavoidable variability arising from factors not fully controlled by the Standard Method, including scan orientation, small differences in numerical computation, and minor implementation differences.

To facilitate this validation, a [^18^F]flortaucipir reference dataset has been made publicly available at (https://www.gaain.org/centaur‐project), enabling sites to process these tau PET scans with their own pipeline and validate their SUVR_Std_ results against those provided at the GAAIN website. Use of this dataset is recommended but not required; investigators may instead validate their pipelines using suitable datasets acquired with other tau PET tracers.

### 3.2 Level B CenTauR computation

Investigators may choose methodological approaches that differ from the Standard Method when alternative acquisition or quantification strategies are better suited to specific research questions, study designs, or available datasets. In such cases, Level B CenTauR computation is required to convert tau PET quantitative metrics derived using non‐standard methods to the CenTauR scale. Non‐standard methods are defined as methods that deviate from any of the steps outlined in Section 2.1 for the Standard Method. These deviations may include the use of non‐standard scanning time windows, quantification pipelines other than the SPM12‐based pipeline, quantification metrics other than SUVR (such as distribution volume ratios [DVR]), or ROIs other than the Standard CenTauR ROIs, including the CenTauR inferior cerebellum GM reference region. Tau PET quantitative data obtained through non‐standard methods cannot be directly converted to the CenTauR scale using the JPM equations in Table 1. Instead, non‐standard tau PET quantitative metrics must first be transformed into SUVR_Std_ to subsequently apply the JPM equations and derive CenTauR values.

For Level B CenTauR computation, we establish that non‐standard tau PET metrics can only be transformed into SUVR_Std_ using linear regression. This implies that:

1. An approximately linear relationship must exist between the non‐standard tau PET metric and SUVR_Std_. This can be assessed through visual inspection of the relationship between the two variables and the examination of the residuals of the linear fit.
2. The correlation between the non‐standard tau PET metric and SUVR_Std_ must show a strong correlation (*R* ^2^ > 0.7, analogous to the Level 2 process for the Centiloid scale). We acknowledge that this threshold is pragmatic rather than empirically optimized for tau PET and may require refinement as additional tracer‐ and pipeline‐specific calibration data become available.
3. To preserve the spatial information encoded in each brain ROI, site‐specific ROIs used in the derivation of tau PET metrics should approximately match the corresponding CenTauR ROIs. For example, a site‐specific tau PET metric measured in a global ROI encompassing the temporal, parietal, and frontal cortices should not be regressed against SUVR_Std_ in the Meta Temporal CenTauR ROI but rather in the Universal CenTauR ROI. As a general guideline, if a site intends to use a specific CenTauR ROI for calibrating its tau PET metric, the site‐specific ROIs should include only brain regions within the brain lobes covered by the calibrating CenTauR ROI.

If a site‐specific tau PET metric (e.g., DVR or other SUVR metric) does not meet the aforementioned requirements 1 through 3, the tau PET metric cannot be harmonized using the CenTauR framework. In these cases, individual sites must redefine the metric to ensure compliance with CenTauR harmonization.

To evaluate the validity of requirements 1 and 2 and derive a linear equation for transforming the non‐standard tau PET metric into SUVR_Std_, sites should measure both metrics in at least 50 subjects. Of these, at least:

- 15 should be Aβ‐negative CU controls,
- 15 should be Aβ‐positive individuals with AD dementia,^38^ and
- 15 should be Aβ‐positive individuals who are either CU or have mild cognitive impairment (MCI).^39^

If these specific subgroup counts cannot be met, alternative distributions of clinical diagnoses are acceptable, provided that the sample spans a sufficiently wide range of SUVR values for the tracer in question. This wide distribution of SUVR values ensures that tau PET data points are spread continuously across a wide range of tau burden, improving the robustness of the linear regression coefficients. To obtain SUVR_Std_ on these 50 or more subjects, sites should perform a Level A CenTauR computation, ideally using the code we provided on the GAAIN website (https://gaain.org/centaur‐project).

While it is always possible to obtain SUVR_Std_ if a site's tau PET scans were acquired using a standard scanning time window, we acknowledge that it may not be possible to derive SUVR_Std_ from scans using a non‐standard window. For example, if a site has collected [^18^F]MK‐6240 scans using only a 70 to 90 minute interval, neither Level A nor Level B CenTauR computations can be performed based solely on these non‐standard tau PET scans. In this case, the site should collect and/or analyze additional [^18^F]MK‐6240 scans (either newly acquired or from an external dataset) acquired over both the non‐standard 70 to 90 minute window and the standard 90 to 110 minute interval. This would allow the derivation of a linear transformation to convert the non‐standard 70 to 90 minute data into SUVR_Std_ (90–110 minutes). If < 50 such scans are available, a valid approach would involve deriving a conversion equation by applying the GAAIN pipeline to the non‐standard 70 to 90 minute scans and regressing the results against SUVR_Std_ from the GAAIN dataset. This is precisely the approach we used to transform the 70 to 90 minute [^18^F]MK‐6240 SUVR data from the University of Pittsburgh before inputting it into the JPM (Figure 1). The conversion equation can then be used in the set of ≥ 50 scans acquired with non‐standard time windows to derive a conversion equation between the site's non‐standard data and SUVR_Std_. We, however, recommend minimizing the number of sequential calibration steps whenever possible, as uncertainty from each regression may propagate through subsequent transformations. Direct calibration of the non‐standard metric to SUVR_Std_ is therefore preferred when feasible, particularly when high quantitative precision is required.

Once SUVR_Std_ has been measured in the set of ≥ 50 subjects, a scatter plot of the tau PET metric versus SUVR_Std_ can be used to visually confirm a linear relationship, and simple linear regression can be used to confirm appropriate correlation (*R* ^2 ^> 0.7) and obtain the transformation parameters. The non‐standard tau PET metric can then be converted into SUVR_Std_, and equations from Table 1 can then be applied to transform the new SUVR_Std_ into CenTauRs. We recommend that, when converting non‐standard tau PET data to the CenTauR scale, investigators report a scatter plot of the non‐standard metric (*x* axis) versus SUVR_Std_ (*y* axis) in the specific CenTauR ROI, together with the corresponding regression equation and *R* ^2^ value.

#### 3.2.1 Example 1 of Level B CenTauR computation: Meta Temporal SUVR in the ADNI cohort

The ADNI study acquires [^18^F]flortaucipir PET scans using a non‐standard scanning window of 75 to 105 minutes post‐injection and quantifies [^18^F]flortaucipir SUVR in a Meta Temporal ROI based on a combination of FreeSurfer v7.1.0 and SPM12 segmentations.^40^ Therefore, the resulting SUVR values are not compatible with the definition of Standard SUVR (section 2.1), and the equations provided in Table 1 cannot be used to convert these values into CenTauR units. Instead, Level B CenTauR computation, as described in Section 3.2, is required.

Data used in the preparation of this article were obtained from the ADNI database. ADNI was launched in 2003 as a public‐private partnership, led by Principal Investigator Michael W. Weiner, MD. The primary goal of ADNI has been to test whether serial MRI, PET, other biological markers, and clinical and neuropsychological assessments can be combined to measure the progression of MCI and early AD.

To calibrate ADNI's non‐standard Meta Temporal SUVR following the Level B procedure, we included 981 ADNI participants (including 404 Aβ‐negative CU, 160 Aβ‐positive CU, 170 Aβ‐positive MCI, 56 Aβ‐positive AD dementia, and 191 Aβ‐negative cognitively impaired (CI) [MCI or dementia]) who underwent [^18^F]flortaucipir PET and for whom ADNI Meta Temporal SUVR values were available. Because both the ADNI Meta Temporal ROI and the CenTauR Meta Temporal ROI include medial and lateral aspects of the temporal lobe, the natural Standard ROI for calibration is the CenTauR Meta Temporal ROI (Figure 4A, left panel). To make ADNI [^18^F]flortaucipir PET images compatible with the Standard scanning window, we realigned and averaged the 80 to 100 minute frames to create static images. These images were then smoothed using the same kernels applied in the original ADNI harmonization protocol, yielding an effective resolution of 6 mm. Finally, the Standard SPM12‐based quantification pipeline was applied to compute SUVR_Std_, and the results were regressed against the non‐standard ADNI Meta Temporal SUVR (Figure 4A, right panel), confirming a strong linear relationship (*R* ^2^ = 0.98) suitable for CenTauR harmonization. The resulting calibration equation is:

$$SUV\;R_{Std}=1.147\times SUVR_{ADNI\;Meta\;Temporal}^{FTP}-0.110$$

**FIGURE 4:**
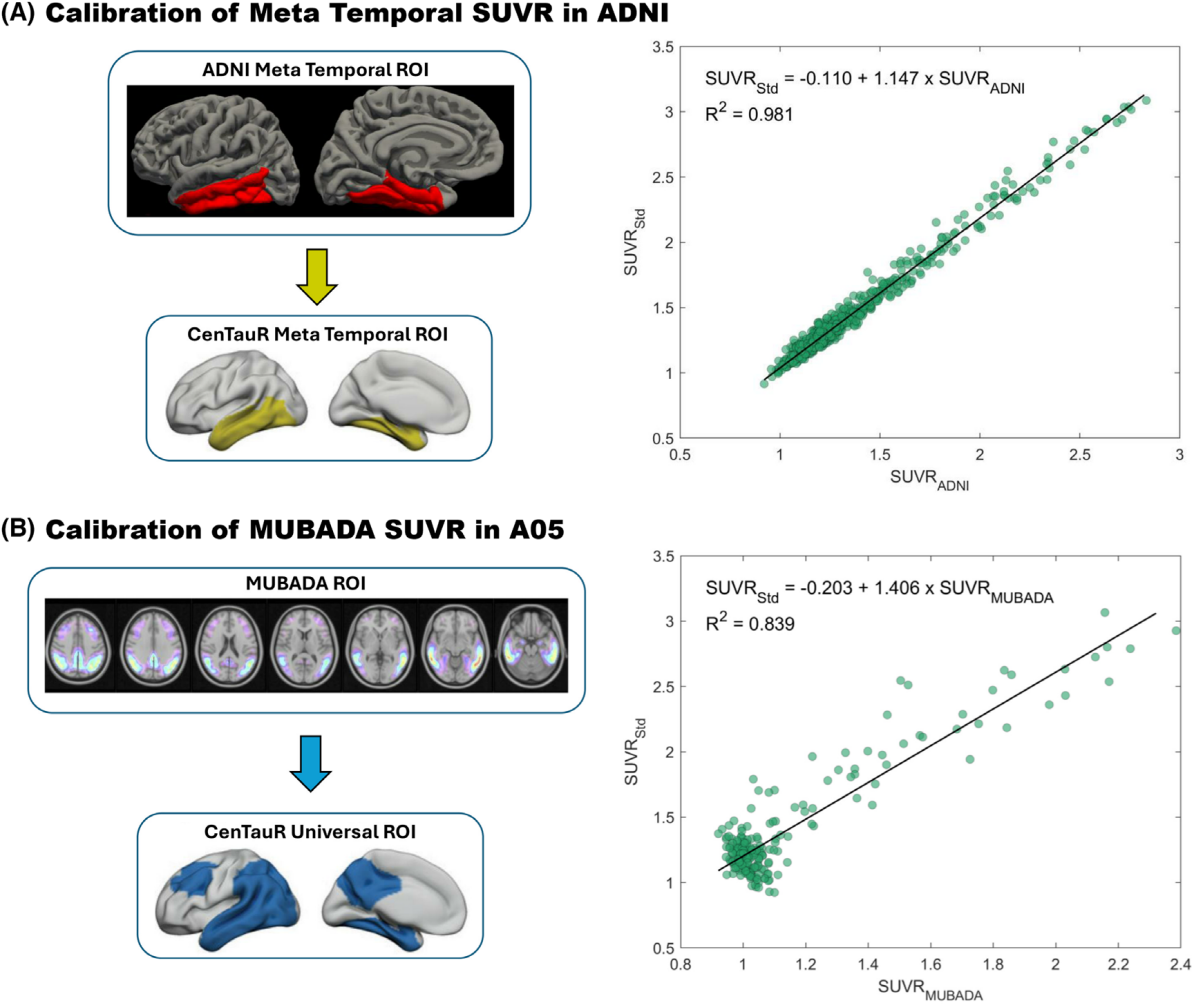
Calibration of non‐standard tau PET metrics to the CenTauR scale. Left panels (upper and lower) illustrate the correspondence between each non‐standard ROI—(A) the ADNI Meta Temporal ROI and (B) the MUBADA ROI—and the matched CenTauR ROI used for calibration. Right panels (upper and lower) show the linear regression models used to convert the non‐standard tau PET metrics—(A) SUVR_ADNI_ and (B) SUVR_MUBADA_ to “Standard” SUVR (SUVR_Std_). The equations displayed in the right panels provide the transformation required to map each non‐standard metric onto SUVR_Std_. ADNI, Alzheimer's Disease Neuroimaging Initiative; MUBADA, multiblock barycentric discriminant analysis; PET, positron emission tomography; ROI, region of interest; SPM, Statistical Parametric Mapping; SUVR, standardized uptake value ratio.

Hence, to convert ADNI's Meta Temporal SUVR values into CenTauR units, this calibration equation is combined with the corresponding CenTauR transformation equation in Table 1 for the CenTauR Meta Temporal ROI, resulting in the final conversion equation into the CenTauR scale:

$$CT\;R_{Meta\;Temporal}=116.55\times SUVR_{ADNI\;Meta\;Temporal}^{FTP}-133.38$$

#### 3.2.2 Example 2 of Level B CenTauR computation: multiblock barycentric discriminant analysis SUVR in the A05 study

The quantification of [^18^F]flortaucipir SUVR in the previous A05 study^10^ was performed using a custom global target ROI that covered temporal, parietal, and frontal cortices (multiblock barycentric discriminant analysis [MUBADA] ROI) and a WM reference region (parametric estimation of reference signal intensity [PERSI]).^41^ The MUBADA SUVR with PERSI reference region (SUVR_MUBADA_) is therefore a non‐standard tau PET metric and it is necessary to transform it into SUVR_Std_ before applying the JPM‐based equations from Table 1. Because the MUBADA ROI spans temporal, parietal, and frontal regions, the most appropriate CenTauR ROI for harmonization is the Universal ROI (Figure 4B, left panel). To derive the conversion equation, we identified 217 subjects (including 67 Aβ‐negative CU, 47 Aβ‐positive MCI, 32 Aβ‐positive AD dementia, and 71 Aβ‐negative CI from the A05 study with previously measured SUVR_MUBADA_. Because all the [^18^F]flortaucipir scans in A05 were acquired with a standard scanning window (80–100 minutes), we can process their [^18^F]flortaucipir scans according to the Standard Method using the SPM12‐based Standard pipeline and derive SUVR_Std_. The relationship between SUVR_MUBADA_ and SUVR_Std_ in the Universal CenTauR ROI is shown in Figure 4B, right panel, showing a strong (*R* ^2^ = 0.84) linear correlation. The derived calibration equation is:

$$SUV\;R_{Std}=1.406\times FTP\;SUVR_{MUBADA}-0.203$$

This equation can be combined with [^18^F]flortaucipir's equation for the Universal CenTauR ROI (Table 1), resulting in the final equation to convert SUVR_MUBADA_ into CenTauRs:

$$CTR=146.10\times FTP\;SUVR_{MUBADA}-144.63$$

## 4 RELEVANT tau PET CUT‐POINTS EXPRESSED IN THE CenTauR SCALE

A major strength of the CenTauR scale is that it enables the direct use of tau PET cut‐points derived from different radiotracers and quantification pipelines across external studies. Below, we summarize key cut‐points expressed in the CenTauR scale that are relevant for various clinical and research applications.

### 4.1 [^18^F]Flortaucipir FDA‐approved visual reads

To date, the only FDA approved tau‐PET radiotracer with an available clinically applicable visual interpretation method is [^18^F]flortaucipir. A positive [^18^F]flortaucipir visual read reflects Braak stages V and VI and high AD neuropathologic changes,^5^ and is strongly associated with clinical progression in preclinical AD as well as in individuals with MCI or dementia.^42^ Despite the importance of clinically applicable methods for establishing tau PET positivity reflecting underlying Braak V and VI stage, there is currently no universally accepted SUVR cut‐point that directly aligns with [^18^F]flortaucipir visual interpretation.

To address this gap, we performed Level A CenTauR computation in 385 CI ADNI participants (MCI or AD dementia) who underwent [^18^F]flortaucipir PET and had FDA‐approved visual reads available, performed by an expert reader (A.M.R.).^42^ Receiver operating characteristic (ROC) analyses were performed using CenTauR values from each ROI as continuous predictors of visual‐read status. Among all CenTauR ROIs, the Meta Temporal ROI provided the strongest discrimination between visually positive and negative scans (area under the curve [AUC] = 0.96; Figure 5) and was therefore selected as the preferred ROI for alignment with visual reads. The optimal threshold was then determined by maximizing the Youden index (*sensitivity + specificity − 1*), resulting in a CenTauR cut‐point of 17.9 for distinguishing visually positive from visually negative scans. Importantly, this CenTauR cut‐point can be applied to tau radiotracers other than [^18^F]flortaucipir, allowing positivity to be interpreted in terms of the neuropathologic changes and clinical outcomes associated with [^18^F]flortaucipir visual reads.

**FIGURE 5:**
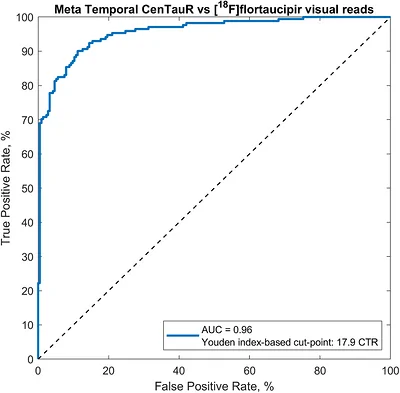
ROC curve analysis of CenTauR Meta Temporal SUVR for classifying FDA‐approved [^18^F]flortaucipir visual reads. The blue line represents the ROC curve, and the red cross indicates the point at which the Youden index is maximized (17.9 CTR). AUC denotes the area under the curve, and PPA and NPA represent positive and negative percent agreement, respectively, at the Youden‐based cut‐point of 17.9 CTR. CTR, CenTauR units; FDA, US Food and Drug Administration; ROC, receiver operating characteristic; SUVR, standardized uptake value ratio.

### 4.2 Biological disease staging

An in vivo tau PET–based biological staging system for AD pathology was recently introduced in the Revised Criteria for Diagnosis and Staging of Alzheimer's Disease.^43^ This system defines four stages within Aβ‐positive individuals, with higher stages reflecting the spatial progression of tau PET retention elevations from medial temporal regions into neocortical areas. However, the proposed framework does not establish the quantitative cut‐points or the precise brain ROIs required to operationalize these stages in practice. A recent large study using the Mayo Clinic Study of Aging and the Mayo Clinic Alzheimer's Disease Research Center addressed this gap by implementing the biological staging system using [^18^F]flortaucipir PET as well as Standard CenTauR ROIs and cut‐points defined in the previous CenTauR_z_ scale (Mesial Temporal: 2.9 CTR_z_; Temporoparietal – Moderate: 3.4 CTR_z_; Temporoparietal – High: 8.3 CTR_z_).^44^ This approach relies on *z* scores referenced to an Aβ‐negative cognitively unimpaired group to standardize tau PET quantification. These CenTauR_z_ cut‐points can be readily translated onto the current CenTauR scale by inverting the previously published CenTauR_z_ conversion equations^23^ and the SPM8‐to‐SPM12 conversion equations available on the CenTauR GAAIN website (https://gaain.org/centaur‐project), yielding the following CenTauR cut‐points: Mesial Temporal: 39.0 CTR; Temporoparietal – Moderate: 25.5 CTR; Temporoparietal – High: 62.8 CTR. The use of these CenTauR cut‐points enables accurate implementation of the proposed staging system across different tau radiotracers and quantification pipelines, while maintaining the intended biological distinctions between stages.

We note that because CenTauR values are region specific, the same underlying level of tau pathology may correspond to different CenTauR cut‐points across ROIs. This reflects differences in anchor point SUVR distributions across different brain regions rather than a lack of harmonization. Accordingly, ROI‐specific thresholds are necessary to preserve the spatial information inherent to tau PET.

### 4.3 Low, medium, and high tau categories

The TRAILBLAZER‐ALZ 1 and 2 trials stratified participants into low, medium, and high tau‐burden groups, with baseline tau group shown to be a meaningful predictor of therapeutic response.^45^, ^46^ This stratification was based on quantitative [^18^F]flortaucipir SUVR_MUBADA_ cut‐points (1.10 and 1.46),^45^ as described in Section 3.2.2. Using the CenTauR conversion equation provided in Section 3.2.2, these thresholds can now be expressed directly on the CenTauR scale, corresponding to 16.1 CTR and 68.7 CTR in the Universal ROI for the low‐ and high‐tau categories, respectively. Expressing these thresholds in CenTauR units enables consistent application of the TRAILBLAZER‐ALZ program's tau classifications across tracers and pipelines.

Table 2 summarizes the CenTauR cut‐points described above, along with their corresponding ROIs and intended applications.

**TABLE 2:** Relevant tau PET cut‐points expressed on the CenTauR scale, alongside the corresponding CenTauR brain regions and applications.

Application	CenTauR ROI	CenTauR cut‐point
Classification between negative/positive FDA‐approved [^18^F]flortaucipir visual reads^5^	Meta temporal	17.9
Biological disease staging^44^	Mesial temporal	39.0
	Temporoparietal	Moderate – 25.5
		High – 62.8
Classification into low, medium, and high tau groups^45^	Universal	Low – 16.1
		High – 68.7

Abbreviations: FDA, US Food and Drug Administration; PET, positron emission tomography; ROI, region of interest.

We note that the primary objective of this section and the cut‐points in Table 2 is to provide a practical range of cut‐points to support interpretation of the CenTauR scale, rather than to define definitive thresholds. Although these cut‐points can be applied in other studies with different tracers and be interpreted consistently, we anticipate that they may be refined over time. We therefore encourage the adoption of updated cut‐points as more robust estimates emerge from future studies with optimized designs for cut‐point derivation.

## 5 EXTERNAL VALIDATION OF THE CenTauR APPROACH

After formally defining the steps for implementing the CenTauR harmonization approach and expressing relevant cut‐points in CenTauR units, we conducted an initial evaluation of its performance in external tau PET datasets. For this, we hypothesized that CenTauR harmonization would result in similar frequencies of CenTauR‐defined tau‐PET positivity and biological disease stages across sufficiently comparable cohorts scanned with different radiotracers. To test this hypothesis, we aggregated tau PET scans from external studies to form four separate cohorts, each scanned with either [^18^F]flortaucipir (ADNI,^47^ NACC Standardized Centralized Alzheimer's and Related Dementias Neuroimaging [SCAN] initiative [https://scan.naccdata.org/], and the A05 study^10^, ^48^), [^18^F]MK‐6240 (Chinese Preclinical Alzheimer's Disease Study [C‐PAS]^49^ and SCAN), [^18^F]PI‐2620 (Health & Aging Brain Study Health Disparities [HABS‐HD],^50^ Ludwig Maximilian University [LMU] Hospital,^51^ and SCAN), or [^18^F]RO948 (BioFINDER‐2 [NCT03174938] and Alzheimer's and Families [ALFA+] study^52^). These cohorts were 1:1 matched based on age, Aβ status, and clinical diagnosis (CU, MCI, or clinically diagnosed AD dementia), resulting in a total of 1968 participants (492 per matched cohort scanned with a different tau PET tracer). The characteristics of the included participants are reported in Table S6 in supporting information.

All tau PET scans were acquired using a Standard scanning window (Table S1), with the exception of the [^18^F]PI‐2620 scans obtained at LMU Hospital, which used a non‐standard 30 to 60 minute window and were quantified with an in‐house MR‐less pipeline. For all non‐LMU scans, we processed the data using the SPM12‐based Standard quantification pipeline (https://gaain.org/centaur‐project) to derive CenTauR units. For LMU scans, we calibrated the non‐standard SUVR values (Supplementary Methods 1 in supporting information) according to Level B CenTauR computation (section 3.2) to obtain CenTauR values.

Tau PET positivity was studied using the Meta Temporal ROI cut‐point of 17.9 CTR derived in Section 4.1, corresponding to a visually positive [^18^F]flortaucipir scan. Biological disease staging was implemented using the Alzheimer's Association Revised Criteria framework^43^ consisting of a four‐stage system based on the topography and level of tau deposition (A+T_2_–, A+T_2MTL_+, A+T_2MOD_+, and A+T_2HIGH_+), and cut‐points established as described in Section 4.2 (Mesial Temporal CTR = 39.0, Temporoparietal – Moderate CTR = 25.5, Temporoparietal – High CTR = 62.8). Last, to assess the added value of CenTauR harmonization relative to approaches commonly used in prior research, we (1) defined tau PET positivity using the previous CenTauR_z_ scale^23^ and a cut‐point of 2 CTR_z_, an approach widely adopted in earlier studies to define tau PET positivity^53^, ^54^ and (2) implemented the biological disease staging using previously proposed CenTauR_z_ cut‐points (Mesial Temporal: 2.9 CTR_z_; Temporoparietal – Moderate: 3.4 CTR_z_; Temporoparietal – High: 8.3 CTR_z_).^44^ Results on the CenTauR scale were compared using this standard approach.

Figure 6A shows the frequencies of tau PET positivity—defined using the harmonized cut‐point of 17.9 CTR in the Meta Temporal ROI—across the matched cohorts scanned with different radiotracers. CenTauR harmonization produced highly consistent positivity rates among Aβ‐negative CU, Aβ‐positive CU, and Aβ‐positive CI individuals, with differences across radiotracers < 4 percentage points. Larger variability was observed in Aβ‐negative CI individuals, although this group had a very small sample size (*n* = 11 per cohort). In contrast, applying the previous CenTauR_z_‐based definition of positivity resulted in substantially larger discrepancies, with differences of up to 18 percentage points in the Aβ‐positive CI group (Figure 6B).

**FIGURE 6:**
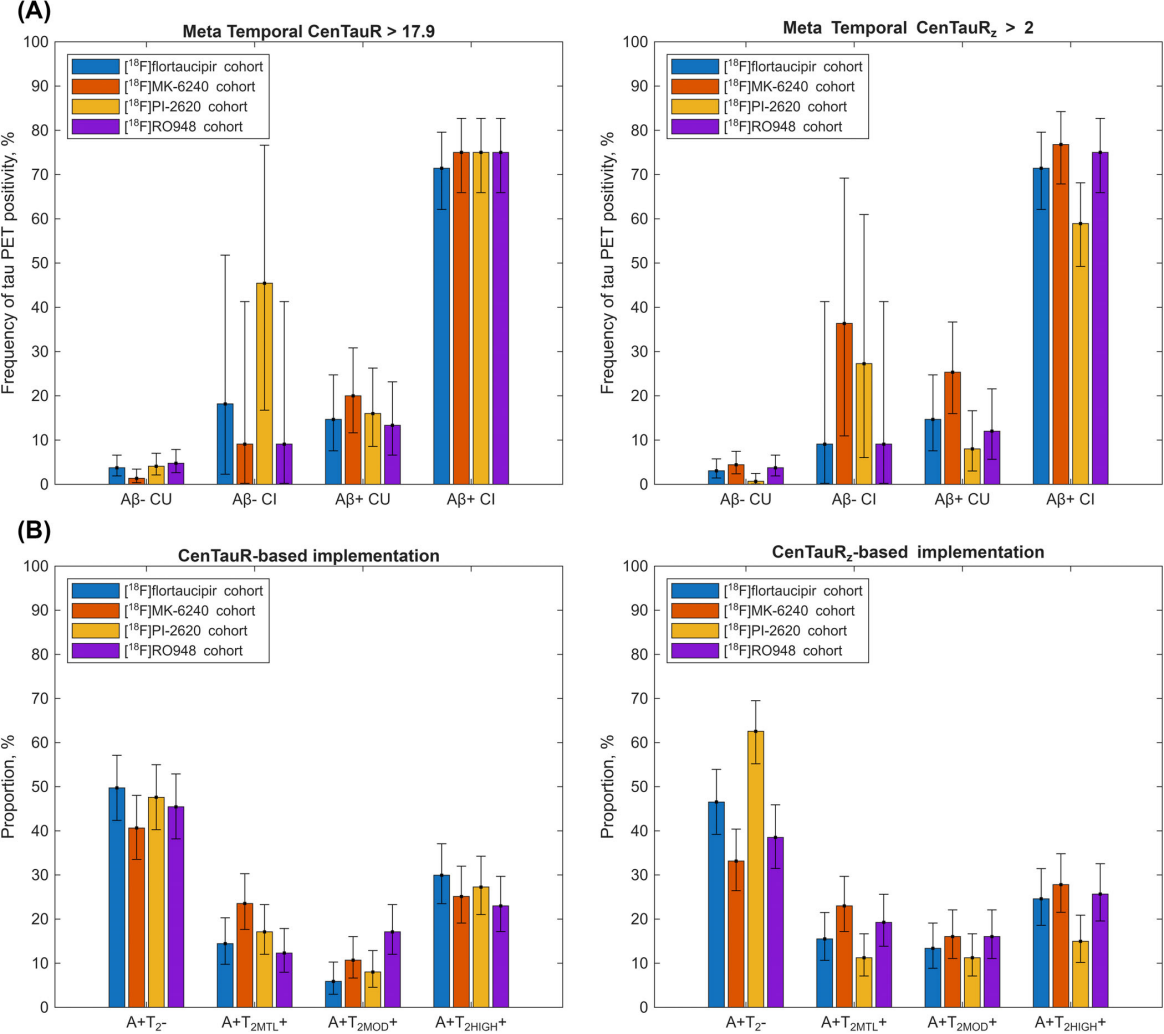
Frequencies of tau‐PET positivity and disease stages using CenTauR. Frequencies of tau PET positivity and biological disease stages across matched cohorts scanned with different tau PET tracers. Panel (A) shows frequencies of tau PET positivity defined using a CenTauR‐based cut‐point (17.9 CTR in the Meta Temporal ROI, corresponding to an FDA‐approved [^18^F]flortaucipir visual read) on the left and a CenTauR_z_‐based cut‐point (2 CTR_z_, a threshold commonly used in prior research) on the right. Panel (B) shows frequencies of biological disease stages defined using CenTauR‐based cut‐points (Mesial Temporal: 39.0 CTR; Temporoparietal – Moderate: 25.5 CTR; Temporoparietal – High: 62.8 CTR) on the left and CenTauR_z_‐based cut‐points (Mesial Temporal: 2.9 CTR_z_; Temporoparietal – Moderate: 3.4 CTR_z_; Temporoparietal – High: 8.3 CTR_z_) on the right. Additional details on the derivation of these cut‐points are provided in Section 4.2. Aβ, amyloid beta; CI, cognitively impaired; CTR, CenTauR units; CU, cognitively unimpaired; FDA, US Food and Drug Administration; PET, positron emission tomography.

Similarly, the frequencies of the different biological stages within the subset of Aβ‐positive individuals (*n* = 187 per cohort) were remarkably consistent when using CenTauR‐based cut‐points, with differences across radiotracers not exceeding 14% in any group (Figure 6A). In contrast, the CenTauR_z_‐based implementation showed substantially greater variability, particularly in the A+T_2_– group, in which differences ranged from 13% to 29% across cohorts (Figure 6B).

## 6 DISCUSSION

Here, we culminate previous efforts on tau PET standardization^23^, ^24^ and formally establish the CenTauR approach, a standardized framework for quantifying tau PET imaging data across different radiotracers and analysis pipelines. Similar to the Centiloid scale for Aβ PET,^22^ the main aim of the CenTauR method is to provide a common unit of measurement, facilitating direct comparisons of tau PET quantitative results across studies and enabling the use of externally derived reference values. To support broad adoption of the CenTauR method, we introduced two distinct approaches for CenTauR computation: (1) direct computation using the Standard Method (Level A CenTauR computation), with publicly available code for implementation (https://gaain.org/centaur‐project); and (2) transformation of site‐specific tau PET quantitative data into the CenTauR scale (Level B CenTauR computation). In addition, calibration datasets and SUVR_Std_ values for multiple tracers have been made available through GAAIN to facilitate calibration of non‐standard pipelines. A pilot evaluation of the CenTauR approach in external matched datasets acquired with different radiotracers demonstrates improved comparability of tau PET metrics, with small between‐tracer differences in the frequency of CenTauR‐defined tau PET positivity and biological disease stages. Importantly, CenTauR harmonization demonstrated substantial improvement over commonly used *z* score–based methods (CenTauR_z_). Together, these findings support the feasibility, reliability, and added value of the CenTauR approach for harmonizing tau PET imaging, enhancing cross‐study comparability, and enabling more consistent interpretation of tau PET data in research and clinical trials.

Although the development of the CenTauR scale is strongly inspired by the Centiloid method, there are key differences that reflect methodological advancements in the statistical frameworks used for harmonization and the unique characteristics of tau PET imaging. One major distinction is the use of the JPM for deriving conversion equations into the CenTauR scale, which eliminates the need for a reference radiotracer. The reliance on a reference tracer in tau PET imaging would present unresolved challenges, including the lack of clear criteria for determining which radiotracer is best suited as the reference standard. Another key difference is that the CenTauR approach was designed to support both regional and global tau burden harmonization. This ensures compatibility not only with dichotomous definitions of tau PET positivity but also with topography‐based tau staging frameworks, extending the applications of CenTauR beyond those of the Centiloid scale, which was limited to the harmonization of global burden metrics.

A key assumption of the CenTauR approach is that the relationship between SUVR values measured across different tracers can be modeled as a linear function. While this assumption may not always hold perfectly, the downsides of introducing non‐linear transformations may outweigh the benefits. In clinical trials, applying non‐linear transformations to outcome data from a single tracer can distort change‐from‐baseline analyses by altering the scale and interpretation of differences.^55^, ^56^, ^57^ These transformations may introduce baseline dependency, disrupt additivity, and ultimately affect statistical significance. Consequently, non‐linear transformations can lead to different conclusions compared to using SUVR as the outcome metric, which is an undesirable property for a harmonization strategy. By using linear conversion equations, the CenTauR approach ensures that statistical results remain unchanged regardless of whether CenTauR values or SUVR data are used. Our initial analysis of external matched cohorts, demonstrating highly consistent tau PET positivity and biological stage frequencies across tracers, further supports the minimal impact of not explicitly modeling non‐linearities. Yet, it is possible that linear transformations may be less accurate at lower cut‐points; however, the biological meaningfulness and clinical relevance of such thresholds remain to be established in appropriate PET‐to‐autopsy studies. Ultimately, these studies will be essential to determine whether the added complexity of non‐linear transformations is justified by a more accurate description of the underlying pathology.

It is important to clarify the interpretation of the CenTauR scale anchor at CTR = 100. In contrast to the Centiloid framework—in which the upper anchor reflects typical late‐onset AD—the CTR = 100 anchor was intentionally defined using younger AD dementia patients (age < 75 years). This choice was motivated by evidence that younger patients—particularly those with early‐onset AD dementia—exhibit higher tau PET signal than older individuals with a comparable degree of cognitive impairment. In analogy with the Centiloid framework, in which the AD dementia anchor corresponds to the upper range of possible Aβ burden, our intention was for CTR = 100 to represent the upper range of tau pathology in AD, and therefore we defined CTR = 100 based on a younger AD dementia population. As a result, CTR = 100 should be interpreted as representing a relatively high tau burden characteristic of younger AD patients, rather than the average signal observed across all AD populations.

Although the datasets used to develop the CenTauR scale were not particularly diverse in terms of race and ethnicity, we expect the framework to remain robust across populations. The CenTauR method is primarily based on head‐to‐head tau PET data and, to date, there is no evidence that demographic factors such as race or ethnicity systematically alter these within‐subject tracer relationships. The limited diversity of the anchor‐point samples is also unlikely to affect the performance of the method, as these samples are used only to define the reference values corresponding to 0 and 100 CenTauRs. Their composition therefore only influences the interpretation of the 0 and 100 values of the scale rather than the validity of the harmonization framework across populations. Nevertheless, we acknowledge that further validation in more demographically diverse cohorts will still be important.

While the CenTauR method represents a significant advancement in standardizing tau PET imaging, several inherent limitations remain, many of which are shared with the Centiloid scale. First, it is unclear how differences in PET scanners, reconstruction algorithms, or image resolution might bias CenTauR quantification. Although the Standard Method harmonizes key aspects of image processing and quantification, residual scanner‐ and reconstruction‐related differences may still affect measured SUVR values and therefore propagate to CenTauR estimates. The magnitude of these effects, and whether they differ across tracers or brain regions, has not yet been systematically evaluated and will require dedicated cross‐scanner and reconstruction studies. Second, the impact of alternative reference regions, such as WM composites commonly used in longitudinal studies, on CenTauR harmonization remains unknown. Third, a potential limitation is that the Standard CenTauR ROIs were defined based on late‐onset AD populations. As tau deposition patterns may differ in early‐onset AD and other clinical or atypical populations, the generalizability of these ROIs—and, consequently, the harmonization framework—may be limited in such groups. Further work is needed to evaluate and potentially adapt the approach across diverse tau topographies. Fourth, although our analyses demonstrated consistent performance of the CenTauR framework across multiple tau PET tracers in matched cohorts, its generalizability requires further independent validation. In particular, future studies should evaluate CenTauR using demographically diverse head‐to‐head datasets that were not included in its development, as well as tracer‐independent reference standards, such as a harmonized visual‐read approach. Such validation will be essential to establish the robustness of the framework across different populations, acquisition settings, and both current and future tau tracers. Importantly, the JPM was designed to facilitate this continued expansion by allowing conversion parameters for newly introduced tracers to be estimated without modifying the conversion equations presented here. Fifth, we reiterate that the proposed CenTauR cut‐points are intended as practical reference values to aid interpretation of the CenTauR scale and may be refined as more robust estimates become available. Sixth, that anchor‐point sample sizes varied across tracers, with some datasets including relatively few participants. However, we do not expect this imbalance to substantially affect the CenTauR transformations because the JPM models all tracers and anchor datasets jointly, with the common 0 and 100 CenTauR reference points informed by the pooled anchor data rather than estimated separately for each tracer. Seventh, the CenTauR Standard Method relies on SUVR rather than DVR or other dynamic PET measures, which may provide more accurate quantification of tau burden. This choice reflects a practical trade‐off between quantitative accuracy and applicability, as SUVR is the predominant metric used in tau PET studies. Requiring dynamic measures would make site‐level calibration considerably more burdensome. Nevertheless, DVR and other dynamic measures can still be harmonized to the CenTauR scale through Level B calibration when an appropriate linear relationship with Standard SUVR exists. Finally, the accuracy of the CenTauR approach in longitudinal studies remains an open question, largely due to the limited availability of longitudinal tau PET data across multiple tracers. Future research will be needed to assess the stability and consistency of the CenTauR scale in longitudinal analyses, although preliminary analyses suggest strong consistency.^58^

## CONFLICT OF INTEREST STATEMENT

Alexis Moscoso Rial and Antoine Leuzy are affiliated to ZRO Imaging. Antoine Leuzy reports consulting services to Enigma Biomedical Group. Sulantha Mathotaarachchi reports employment with Enigma Biomedical Group. Lars Lau Raket, Emily C. Collins, Leonardo Iaccarino, Michael J. Pontecorvo, and Mark A. Mintun report employment with Eli Lilly. Gregory Klein and Matteo Tonietto report employment with F. Hoffmann–La Roche Ltd. Emily Olafson, Sandra Sanabria Bohorquez, and Olivia Lutz report employment with Genentech, Inc. Ziad S. Saad reports employment with Janssen. Roger N. Gunn reports employment with XingImaging, and Alex Whittington reports employment with Perceptive Discovery. Santiago Bullich, Andrew W. Stephens, and Guilherme Domingues Kolinger report employment with Life Molecular Imaging (now part of Lantheus). Arnaud Charil and Michael C. Irizarry report employment with Eisai Inc. Jessica A. Collins and R. Matthew Hutchison report employment with Biogen. Eric Hostetler reports employment with Merck & Co., Inc. Maria C. Carrillo reports employment with the Alzheimer's Association. Yashmin Karten reports employment with the Critical Path Institute (CPAD). Billy Dunn reports advisory roles with Arch Venture Partners, Loulou Foundation, Cerveau Technologies, the Michael J. Fox Foundation, the Epilepsy Foundation, and F‐Prime Capital, and leadership roles with the Virginia Neurological Society and Prothena, Inc. Tobey J. Betthauser reports NIH/NIA grant support (R01AG080766) and honoraria and travel support from academic and research institutions. Christopher C. Rowe reports research support from the National Health and Medical Research Council of Australia, Eisai, and Roche; consulting and advisory roles with Eisai, Eli Lilly, Novo Nordisk, and Prothena; and honoraria for lectures from multiple industry sponsors. Christopher G. Schwarz reports grant support from NIH and consulting fees from Splink, Inc. Cécile Tissot reports consulting fees from Genentech unrelated to the present work. Brian J. Lopresti reports grant support from NIA (P01 AG025024). Victor L. Villemagne reports research funding from NHMRC, the Aging Mind Foundation, and NIH, and consulting or speaking fees from Eli Lilly, Life Molecular Imaging, AC Immune, and other organizations. Makoto Higuchi reports research funding from JST and AMED and holds patents related to tau PET tracers licensed to APRINOIA Therapeutics. Gil D. Rabinovici reports research support from Avid Radiopharmaceuticals, GE Healthcare, Life Molecular Imaging, and Genentech, and consulting fees from Alector, Eli Lilly, Johnson & Johnson, and Merck. Ruben Smith reports speaker fees from Roche. Oskar Hansson reports institutional research support from multiple industry partners and personal consultancy or speaker fees from AC Immune, BioArctic, Biogen, Cerveau, Eisai, Eli Lilly, Fujirebio, Merck, Roche, and others. Marta Marquié reports research funding from Instituto de Salud Carlos III and travel support and advisory roles with industry partners. Mercè Boada reports consultancy fees from multiple pharmaceutical companies. Keith A. Johnson reports consulting fees from Novartis and Merck. Sterling C. Johnson reports consulting fees from Enigma Biomedical Group and Alzpath. All other authors report no competing interests. Author disclosures are available in the Supporting Information.

## CONSENT STATEMENT

All participants provided written informed consent, and each contributing study was approved by the relevant local institutional review boards or ethics committees in accordance with the Declaration of Helsinki.

## Supporting information



Supporting Information

Supporting Information
