# Supplementary material for: Tau positron emission tomography harmonization using the CenTauR approach: General conversion equations, relevant cut‐points, and external validation
Source: Alzheimers Dement. 2026 Oct 30;22(10):e71886. doi: 10.1002/alz.71886 (PMC13722786; doi:10.1002/alz.71886)
Supplement: Supplementary file 2 [file ALZ-22-e71886-s001.docx]

**Supplementary Material**

**Supplementary Figure 1.** Relationship between Standard SUVR values across five head-to-head datasets comprising participants scanned with two different tau PET tracers, shown for the different Standard CenTauR regions of interest. The red dashed line represents the linear regression fit, whereas the blue solid line represents the smooth-transition piecewise linear model:

$$SUVR_{1} = \beta₀ + \beta₁(SUVR_{2} - k) + \beta₂(SUVR_{2}- k) \cdot½ [1 + \tanh((SUVR_{2} - k) / w)] + \varepsilon$$

where $k$denotes the transition point and $w$controls the smoothness of the transition. The inset reports the gain in Akaike Information Criterion (AIC), defined as $AIC_{\text{linear}}-AIC_{\text{piecewise}}$; positive values therefore indicate improved fit of the piecewise model relative to the linear model.

**Supplementary Figure 2.** Relationship between [¹⁸F]MK-6240 SUVR values computed using the Standard CenTauR pipeline with a 70–90 minute acquisition window versus the standard 90–110 minute acquisition window.

**Supplementary Figure 3.** Test-retest tau-PET SUVR data for each radiotracer included in the derivation of the Joint Propagation Model’s CenTauR conversion equations.

**Supplementary Table 1.** Standard scanning time windows for each tau-PET radiotracer.

| Radiotracer | Scanning time window |
| --- | --- |
| [^18^F]Flortaucipir | 80–100 min |
| [^18^F]MK-6240 | 90–110 min |
| [^18^F]PI-2620 | 45–75 min |
| [^18^F]RO948 | 70–90 min |
| [^18^F]GTP-1 | 60–90 min |

**Supplementary Table 2.** Head-to-head, anchor-point, and test-retest datasets used for derivation of the general CenTauR conversion equations

| **Dataset type** | **Radiotracer(s)** | **Cohort / data source** | **Sample size** | **Reference** |
| --- | --- | --- | --- | --- |
| Head-to-head | [¹⁸F]flortaucipir vs [¹⁸F]MK-6240 | University of Pittsburgh | n = 15 | Gogola et al., 2022 [1] |
| Head-to-head | [¹⁸F]flortaucipir vs [¹⁸F]RO948 | BioFINDER-2 | n = 34 | Smith et al., 2020 [2] |
| Head-to-head | [¹⁸F]MK-6240 vs [¹⁸F]GTP1 | Roche / Invicro | n = 22 | Olafson et al., 2025 [3] |
| Head-to-head | [¹⁸F]PI-2620 vs [¹⁸F]GTP1 | Roche / Invicro | n = 28 | Olafson et al., 2025 [3] |
| Head-to-head | [¹⁸F]PI-2620 vs [¹⁸F]RO948 | FACEHBI | n = 17 | Tonietto et al., 2024 [4] |
| Anchor point | [¹⁸F]flortaucipir | Avid A05 | CTR-0: n = 59; CTR-100: n = 31 | Pontecorvo et al., 2019 [5] |
| Anchor point | [¹⁸F]MK-6240 | AIBL | CTR-0: n = 94; CTR-100: n = 63 | Krishnadas et al., 2023 [6] |
| Anchor point | [¹⁸F]PI-2620 | Life Molecular Imaging / ADNI | CTR-0: n = 10; CTR-100: n = 7 | LMI: Mueller et al., 2020 [7]  ADNI: Jagust et al., 2015 [8] |
| Anchor point | [¹⁸F]RO948 | BioFINDER-2 | CTR-0: n = 34; CTR-100: n = 29 | Leuzy et al., 2020 [9] |
| Anchor point | [¹⁸F]GTP1 | Roche / Invicro | CTR-0: n = 15; CTR-100: n = 25 | Sanabria Bohórquez et al., 2019 [10] |
| Test-retest | [¹⁸F]flortaucipir | Avid | n = 21 | Devous et al., 2018 [11] |
| Test-retest | [¹⁸F]MK-6240 | Takeda | n = 15 | Salinas et al., 2020 [12] |
| Test-retest | [¹⁸F]PI-2620 | Life Molecular Imaging | n = 12 | Bullich et al., 2020 [13] |
| Test-retest | [¹⁸F]RO948 | Roche / Invicro | n = 9 | Kuwabara et al., 2018 [14] |
| Test-retest | [¹⁸F]GTP1 | Roche / Invicro | n = 20 | Sanabria Bohórquez et al., 2019 [10] |

**Supplementary Table 3.** Demographic and biomarker characteristics of the subjects included in the head-to-head datasets.

|  | Diagnostic groups | | | |  |
| --- | --- | --- | --- | --- | --- |
|  | CU | MCI | AD dementia | |  |
| [^18^F]flortaucipir vs [^18^F]MK-6240 | | | | |  |
| N | 9 | 1 | 5 | |  |
| Age, y (SD) | 76.4 (3.9) | 77 | 70.8 (13.7) | |  |
| Sex (M/F) | 1/8 | 1/0 | 1/4 | |  |
| MMSE (SD) | 28.2 (3.1) | 22 | 19.2 (7.6) | |  |
| Amyloid positive, n (%) | 5 (56%) | 1 (100%) | 5 (100%) | |  |
| [^18^F]flortaucipir vs [^18^F]RO948 | | | | |  |
| N | 5 | 1 | 5 | |  |
| Age, y (SD) | 77.5 (6.4) | 77 | 70.80 (13.70) | |  |
| Sex (M/F) | 3/2 | 1/0 | 1/4 | |  |
| MMSE (SD) | 29.31 (0.69) | 22 | 19.20 (7.63) | |  |
| Amyloid positive, n (%) | 2 (40%) | 1 (100%) | 5 (100%) | |  |
| [^18^F]MK-6240 vs [^18^F]GTP-1 | | | | |  |
| N | 5 | 3 | 14 | |  |
| Age, y (SD) | 68.0 (2.5) | 73.7 (4.0) | 71.6 (7.9) | |  |
| Sex (M/F) | 1/4 | 1/2 | 7/7 | |  |
| MMSE (SD) | 29.8 (0.4) | 27.6 (1.1) | 17.9 (4.0) | |  |
| Amyloid positive, n (%) | 1 (20%) | 3 (100%) | 14 (100%) | |  |
| [^18^F]PI-2620 vs [^18^F]RO948 |  |  |  |  | |
| N | 13 | 5 | NA | |  |
| Age, y (SD) | 73 (7) | 77 (3) | NA | |  |
| Sex (M/F) | 7/6 | 2/3 | NA | |  |
| MMSE (SD) | 29.62 (0.65) | 28.20 (1.30) | NA | |  |
| Aβ-positive, n (%) | 7 (54%) | 2 (40%) | NA | |  |
| [^18^F]PI-2620 vs [^18^F]GTP-1 |  |  |  |  | |
| N | 5 | 10 | 12 | |  |
| Age, y (SD) | 71 (1.0) | 72 (5.4) | 74 (3.0) | |  |
| Sex (M/F) | 4/1 | 4/6 | 7/5 | |  |
| MMSE (SD) | 30.0 (0.0) | 26.9 (1.8) | 22.5 (4.4) | |  |
| Amyloid positive, n (%) | 0 (0%) | 10 (100%) | 12 (100%) | |  |

**Supplementary Table 4.** Demographic and biomarker characteristics of the subjects included in the anchor point datasets.

|  | [^18^F]flortaucipir | [^18^F]MK-6240 | [^18^F]PI-2620 | [^18^F]RO948 | [^18^F]GTP-1 |
| --- | --- | --- | --- | --- | --- |
|  |  |  |  |  |  |
| N |  |  |  |  |  |
| CTR-0 | 59 | 94 | 10 | 34 | 15 |
| CTR-100 | 31 | 63 | 7 | 29 | 25 |
| Age, y (SD) |  |  |  |  |  |
| CTR-0 | 57.4 (19.0) | 71 (3) | 59.2 (7.2) | 51.5 (2.3) | 64.0 (6.9) |
| CTR-100 | 66.2 (6.9) | 66.3 (6.5) | 61.2 (6.8) | 63.2 (4.4) | 67.0 (5.9) |
| Sex (M/F) |  |  |  |  |  |
| CTR-0 | 30/29 | 38/56 | 6/4 | 13/21 | 8/7 |
| CTR-100 | 15/16 | 35/38 | 4/3 | 13/16 | 13/12 |
| MMSE (SD) |  |  |  |  |  |
| CTR-0 | 29.5 (0.5) | 29.2 (0.9) | 29.0 (1.2) | 29.2 (1.2) | 29.5 (0.8) |
| CTR-100 | 23.7 (2.0) | 23.3 (2.3) | 22.6 (2.7) | 22.4 (4.2) | 23.3 (2.1) |
| Aβ-PET, Centiloids |  |  |  |  |  |
| CTR-0 | -2 (7) | -2 (6) | -2 (6) | -12 (5) | 0 (11) |
| CTR-100 | 99 (29) | 113 (26) | 96 (29) | 96 (20) ^†^ | 98 (27) |

^†^Amyloid PET data were available for 75 of 96 participants; for the remaining 21 participants, amyloid positivity was determined using CSF Aβ42/40.

**Supplementary Table 5.** Demographic and biomarker characteristics of the subjects included in the test-retest datasets.

|  | [^18^F]flortaucipir | [^18^F]MK-6240 | [^18^F]PI-2620 | [^18^F]RO948 | [^18^F]GTP-1 |
| --- | --- | --- | --- | --- | --- |
|  |  |  |  |  |  |
| N | 23 | 15 | 6 | 9 | 20 |
| Age, y (SD) | 70.87 (7.78) | 63 (5.0) | 66 (6.4) | 62.3 (6.50) | 68 (8.10) |
| Sex (M/F) | (15/8) | 5/10 | 3/3 | 8/1 | 9/11 |
| Clinical diagnosis, N |  |  |  |  |  |
| CU | 5 | 3 | 3 | 5 | 5 |
| MCI | 8 | 0 | 0 | 0 | 0 |
| AD dementia | 10 | 12 | 3 | 4 | 15 |
| Aβ-positive, n (%) | NA | 12 (80%) | 3 (50%) | 4 (44%) | 15 (75%) |

**Supplementary Table 6.** Characteristics of the matched tau-PET cohorts.

|  | Cognitively Unimpaired | | |  | Cognitively Impaired (MCI or AD dementia) | | |  |
| --- | --- | --- | --- | --- | --- | --- | --- | --- |
| Cohort | **[^18^F]Flortaucipir (ADNI, A05, SCAN)** | **[^18^F]MK-6240 (CPAS, SCAN)** | **[^18^F]PI-2620 (HABS-HD, LMU, SCAN)** | **[^18^F]RO948 (BioFINDER-2, ALFA+)** | **[^18^F]Flortaucipir (ADNI, A05, SCAN)** | **[^18^F]MK-6240 (CPAS, SCAN)** | **[^18^F]PI-2620 (HABS-HD, LMU, SCAN)** | **[^18^F]RO948 (BioFINDER-2)** |
| N | 369 | 369 | 369 | 369 | 123 | 123 | 123 | 123 |
| Age | 70.1 (6.7) | 69.5 (7.2) | 69.1 (7.5) | 69.8 (6.9) | 72.7 (8.0) | 72.1 (7.8) | 72.4 (7.9) | 72.7 (8.0) |
| Aβ-positive N, (%) | 75 (20%) | 75 (20%) | 75 (20%) | 75 (20%) | 112 (91%) | 112 (91%) | 112 (91%) | 112 (91%) |

**Supplementary Methods 1.** Description of calibration of LMU [^18^F]PI-2620 SUVR values for Level-B CenTauR Computation.

The [^18^F]PI-2620 scans from LMU Hospital were acquired using a non-standard 30–60 minute post-injection window. Moreover, because structural T1-weighted scans were unavailable for a substantial proportion of participants, these scans were quantified using an in-house MR-less pipeline consistent with the MR-less quantification procedures currently implemented in SCAN [15]. Briefly, we generated a 30–60 minute [^18^F]PI-2620 template using data from the SCAN study, selecting participants who had both 30-60 minute [^18^F]PI-2620 PET and a corresponding structural T1 scan (n = 34). To construct the template, we first spatially normalized each T1-weighted image using the “Old Normalise” tool in SPM12 and then applied the resulting transformation parameters to the coregistered [^18^F]PI-2620 images. We used this pipeline to spatially normalize LMU [^18^F]PI-2620 scans and compute SUVR in the ‘Standard’ CenTauR ROIs.

Next, to calibrate the non-standard SUVR values, we employed a two-step procedure due to the lack of paired 30–60 and 45–75 minute [^18^F]PI-2620 scans with accompanying structural T1 images in the SCAN dataset. We first ran the MR-less pipeline on 23 subjects who had paired 30–60 and 45–75 minute [^18^F]PI-2620 scans and derived conversion equations from MR-less 30–60 to MR-less 45–75 using linear regression:

Next, we identified another set of 68 SCAN participants with available 45–75 minute [^18^F]PI-2620 scans and structural T1 images and ran both the MR-less pipeline as well as the ‘Standard’ pipeline to derive SUVR_Std_, allowing the derivation of conversion equations from MR-less 45-75 minute to SUVR_Std_:

The combination of these two sets of equations resulted in the following conversion equations to convert LMU Hospital MR-less 30-60 minute SUVR data to SUVR_Std_:

| **CenTauR ROI** | **Conversion equation** |
| --- | --- |
| Meta Temporal | $SUVR_{Std}= 1.21\times SUVR_{MR_{less}30-60}-0.29$ |
| Universal | $SUVR_{Std}= 1.30\times SUVR_{MR_{less}30-60}-0.41$ |
| Mesial Temporal | $SUVR_{Std}= 1.13\times SUVR_{MR_{less}30-60}-0.18$ |
| Temporoparietal | $SUVR_{Std}= 1.32\times SUVR_{MR_{less}30-60}-0.41$ |
| Frontal | $SUVR_{Std}= 1.34\times SUVR_{MR_{less}30-60}-0.44$ |

**Supplementary References**

[1] Gogola A, Minhas DS, Villemagne VL, Cohen AD, Mountz JM, Pascoal TA, et al. Direct Comparison of the Tau PET Tracers (18)F-Flortaucipir and (18)F-MK-6240 in Human Subjects. J Nucl Med. 2022;63:108-16.

[2] Smith R, Scholl M, Leuzy A, Jogi J, Ohlsson T, Strandberg O, et al. Head-to-head comparison of tau positron emission tomography tracers [(18)F]flortaucipir and [(18)F]RO948. Eur J Nucl Med Mol Imaging. 2020;47:342-54.

[3] Olafson E, Tonietto M, Klein G, Teng E, Stephens AW, Russell DS, et al. In Vivo Head-to-Head Comparison of [(18)F]GTP1 with [(18)F]MK-6240 and [(18)F]PI-2620 in Alzheimer Disease. J Nucl Med. 2025;66:277-85.

[4] Tonietto M, Sotolongo-Grau O, Roe-Vellve N, Bullich S, Tartari JP, Sanabria A, et al. Head-to-head comparison of tau PET tracers [(18)F]PI-2620 and [(18)F]RO948 in non-demented individuals with brain amyloid deposition: the TAU-PET FACEHBI cohort. Alzheimers Res Ther. 2024;16:257.

[5] Pontecorvo MJ, Devous MD, Kennedy I, Navitsky M, Lu M, Galante N, et al. A multicentre longitudinal study of flortaucipir (18F) in normal ageing, mild cognitive impairment and Alzheimer's disease dementia. Brain. 2019;142:1723-35.

[6] Krishnadas N, Dore V, Robertson JS, Ward L, Fowler C, Masters CL, et al. Rates of regional tau accumulation in ageing and across the Alzheimer's disease continuum: an AIBL (18)F-MK6240 PET study. EBioMedicine. 2023;88:104450.

[7] Mueller A, Bullich S, Barret O, Madonia J, Berndt M, Papin C, et al. Tau PET imaging with (18)F-PI-2620 in Patients with Alzheimer Disease and Healthy Controls: A First-in-Humans Study. J Nucl Med. 2020;61:911-9.

[8] Jagust WJ, Koeppe RA, Rabinovici GD, Villemagne VL, Harrison TM, Landau SM, et al. The ADNI PET Core at 20. Alzheimers Dement. 2024;20:7340-9.

[9] Leuzy A, Smith R, Ossenkoppele R, Santillo A, Borroni E, Klein G, et al. Diagnostic Performance of RO948 F 18 Tau Positron Emission Tomography in the Differentiation of Alzheimer Disease From Other Neurodegenerative Disorders. JAMA Neurol. 2020;77:955-65.

[10] Sanabria Bohorquez S, Marik J, Ogasawara A, Tinianow JN, Gill HS, Barret O, et al. [(18)F]GTP1 (Genentech Tau Probe 1), a radioligand for detecting neurofibrillary tangle tau pathology in Alzheimer's disease. Eur J Nucl Med Mol Imaging. 2019;46:2077-89.

[11] Devous MD, Sr., Joshi AD, Navitsky M, Southekal S, Pontecorvo MJ, Shen H, et al. Test-Retest Reproducibility for the Tau PET Imaging Agent Flortaucipir F 18. J Nucl Med. 2018;59:937-43.

[12] Salinas C, Lohith TG, Purohit A, Struyk A, Sur C, Bennacef I, et al. Test-retest characteristic of [(18)F]MK-6240 quantitative outcomes in cognitively normal adults and subjects with Alzheimer's disease. J Cereb Blood Flow Metab. 2020;40:2179-87.

[13] Bullich S, Barret O, Constantinescu C, Sandiego C, Mueller A, Berndt M, et al. Evaluation of Dosimetry, Quantitative Methods, and Test-Retest Variability of (18)F-PI-2620 PET for the Assessment of Tau Deposits in the Human Brain. J Nucl Med. 2020;61:920-7.

[14] Kuwabara H, Comley RA, Borroni E, Honer M, Kitmiller K, Roberts J, et al. Evaluation of (18)F-RO-948 PET for Quantitative Assessment of Tau Accumulation in the Human Brain. J Nucl Med. 2018;59:1877-84.

[15] Landau SM, Ward TJ, Murphy A, Iaccarino L, Harrison TM, La Joie R, et al. Quantification of amyloid beta and tau PET without a structural MRI. Alzheimers Dement. 2023;19:444-55.
